# Commentary: Predicting patient deterioration by nurse intuition: the development and validation of the Nurse Intuition Patient Deterioration Scale

**DOI:** 10.3389/fmed.2024.1259449

**Published:** 2024-01-25

**Authors:** Feng Zhao, Jihu Zhao, Heng Liu

**Affiliations:** ^1^School of Nursing, Qingdao University, Qingdao, Shandong, China; ^2^Department of Neurosurgery, The Affiliated Hospital of Qingdao University, Qingdao, Shandong, China

**Keywords:** nurse intuition, patient deterioration, scale development, predict, NEWS

## 1 Introduction

Serious adverse outcomes in patients can be avoided by early detection and response to clinical and physiological deterioration (1), and nurses play an important role in recognizing patient deterioration (2). Nurses typically make decisions based on patient's vital signs, such as heart rate, pulse, and blood pressure, rather than their intuition (3). Recently, Haegdorens et al. developed and validated the Nurse Intuition Patient Deterioration Scale (NIPDS), which was published in the International Journal of Nursing Studies (4). This scale includes nine aspects of patients' conditions (oral expression ability, self-feeling, facial expressions, consciousness, abnormal behavior, skin color, responsiveness, and gaze), which nurses rated using one of the three response categories ranging from zero (not present) to two (very present), with a total score ranging from zero to 18. When the overall score was ≥5, patients were categorized as being at high risk. It demanded additional attention and an immediate response from those equipped to assess and treat critically ill patients. The nurses might have used the NIPDS to identify possible problems in patients even when vital signs did not meet the trigger threshold and other warning tools produced negative results. Although the authors claimed that the NIPDS outperformed the National Early Warning Scale (NEWS) (4), it was disputed whether the scale established on intuition could be used in the clinic. We thus discussed the evaluation indicators in the NIPDS by comparing multiple scales in anticipation of better refining and promoting the NIPDS.

## 2 Review of several warning tools

**Figure 1:**
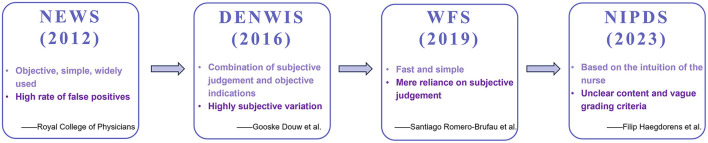
Time–axis plot of various caring scales. NEWS, National Early Warning Scale; DENWIS, Dutch-Early-Nurse-Worry-Indicator-Score; WFS, Worry Factor Score; NIPDS, Nurse Intuition Patient Deterioration Scale.

Previous researchers have created a number of warning measures to detect early deterioration in patients' conditions (Figure 1). The NEWS was recognized as one of the most important clinical decision-making devices based on vital signs and was developed by the Royal College of Physicians (RCP) in the United Kingdom (5, 6). Studies showed that the NEWS had been recommended and implemented to enhance patient safety by grading acute illness severity and detecting patient deterioration (7, 8). Compared to the NIPDS, the NEWS had the following weaknesses. First, the NEWS could not express subtle clinical symptoms or changes in patients, so the scale was inapplicable in some cases where patients might have an acute ailment that did not always significantly interfere with vital signs in relation to their need for urgent care. For example, a patient with acute myocardial infarction might present with a NEWS < 5, but he had other signs and symptoms that required emergency care. Furthermore, the NEWS could generate a large number of false-positive patients and an additional workload for clinicians (9). Another warning tool is the Dutch-Early-Nurse-Worry-Indicator-Score (DENWIS), which is based on the concern of nurses about patients and comprises nine indicators (breathing, circulation, rigors, mentation, agitation, pain, unexpected trajectory, patients' feelings, and nurses' subjective observations) (10). Although studies showed that the DENWIS performed well in predicting unplanned admission to the intensive care unit and unexpected mortality (11, 12), the score indicators might be explained in various ways, so the nurses would cause doubt in the process of assessing. The next 5-level warning tool, the Worry Factor Score (WFS), was graded by the concern of nurses. A score of 0 or 1 indicated that the nurses did not believe the patient was actively deteriorating, but a score >1 suggested that the nurses' concern about patient deterioration had increased (13). This score revealed a great effect in predicting rapid response team calls, transfer to the intensive care unit, and resuscitation calls (13). Nonetheless, the WFS might be assessed differently by nurses if they were working without any clinical clues to guide them. Furthermore, other existing early warning scales, such as the Modified Early Warning Score (MEWS), Rapid Emergency Medicine Score (REMS), Acute Physiology and Chronic Health Evaluation II (APACHE II), Oxford Acute Severity of Illness Score (OASIS), and Multiple Organ Dysfunction Score (MODS), were based on the patient's objective deterioration and ignored the medical staff's subjective judgment (Table 1). Most encouragingly, Haegdorens et al. developed a high-quality and practicable instrument called NIPDS. Nevertheless, the writers might have overlooked something beyond intuition.

**Table 1:** Relevant content of comparable scales.

**Title**	**Indicator**	**Total score**	**Aim**	**Application department**	**Advantage**	**Disadvantage**
MEWS	Heart rate, Systolic blood pressure, Respiration, Temperature, Consciousness	0–14	Assess the condition of patients	ICU, Emergency department	Simple to use, Short time- consuming	High false positive rate
REMS	Blood pressure, Respiration, Pulse, GCS, Age, SpO_2_	0–26	Predict mortality of patients	ICU, Emergency department	High accuracy	Time consuming
APACHE II	Acute Physiology Score, Age, Chronic Health Evaluation	0–71	Assess the condition of patients	ICU, Emergency department	High accuracy, Extensive use	Time consuming
OASIS	Days in hospital, Heart rate, Respiration, Age, Mean arterial pressure, Temperature, GCS, 24 h urine output	0–75	Assess the condition of patients	ICU	Simple to use	Uncertain cutoff value
MODS	PaO_2_, FiO_2_, CVP, GCS, Platelet, Serum creatinine, Serum total bilirubin, Heart rate, Mean arterial pressure	0–24	Predict mortality of patients	ICU	High accuracy	Time consuming

MEWS, Modified Early Warning Score; REMS, Rapid Emergency Medicine Score; APACHE II, Acute Physiology and Chronic Health Evaluation II; OASIS, Oxford Acute Severity of Illness Score; MODS, Multiple Organ Dysfunction Score; GCS, Glasgow Coma Scale; ICU, Intensive Care Unit; CVP, Central Venous Pressure.

## 3 Possible problems with the NIPDS

However, other concerns have to be addressed. First and foremost, the authors did not clearly differentiate between “1-present” and “2-very present.” According to Benner et al., nurse intuition is “a judgment without a rationale, a direct apprehension, and response without recourse to calculative rationality” (14), which cannot be defined and was influenced by factors such as years of work, ward environment, experience, and educational level (15, 16). As a result, if authors are unable to establish objective standards for defining “present” and “very present,” nurses will be unable to use the NIPDS to make appropriate judgments about patients. Second, we believe some items that might confuse nurses need to be updated and enhanced. For instance, the second item's description was inadequate, with no objective indicators. As a result, assessing patients' feelings was challenging, especially for those with communication and consciousness difficulties. Furthermore, in item 7, the change in skin color was a continual process that was not always typical in some circumstances. It was particularly unsuited for the black race and might have been influenced by the natural skin color, making it difficult to observe. The problems mentioned above had the potential to exacerbate the wide difference in final grading results and to impair the judgment of nurses in real-life practice. Additionally, NIPDS measurement data were only acquired from one hospital, so the small sample size may have reduced the accuracy and dependability. The widespread adoption of hospitals in other nations and regions, such as the NEWS, would increase the validity and influence of the scale (17). Finally, the NIPDS validation is insufficient. When evaluating the NIPDS, the writers only used the NEWS. In reality, patient deterioration is assessed using scales such as MEWS, OASIS, MODS, REMS, and APACHE II (Table 1). As a result, the NIPDS should be compared to numerous scales in a variety of clinical situations to provide a more objective and realistic assessment.

## 4 Discussion

Previous studies have shown that vital signs, urine output, and pain are essential indicators of the condition of patients (18, 19). However, the NIPDS was not integrated with the aforementioned indicators. Patients would be at risk of undetected clinical change if vital signs were ignored (20). Of course, it was not advisable to focus only on vital signs and ignore the subjective judgment and intuition of nurses. As a result, we would recommend an approach that combines the NIPDS and the NEWS. In other words, it assesses patient deterioration based on the intuition of nurses and scores numerous physiological markers at the same time. Furthermore, to determine whether the NIPDS was more effective than other scales, the NIPDS should be prospectively validated in other hospitals, healthcare systems, patient categories, and wards as soon as possible during the validation phase. We highly valued the authors' original and novel thoughts. However, using nursing intuition to anticipate patient deterioration requires more research.

## Author contributions

FZ: Writing—original draft. JZ: Writing—original draft, Writing—review & editing. HL: Writing—review & editing.
